# Chromoblastomycosis in Brazil: A review of 450 published cases

**DOI:** 10.1590/0037-8682-0132-2024

**Published:** 2024-11-15

**Authors:** Layala Stefane de Paula Barbosa, Yury Raphaell Coringa de Souza, Caroline Suemi Sasaki, Daniel Wagner dos Santos, Luana Rossato

**Affiliations:** 1 Universidade Federal da Grande Dourados, Faculdade de Ciências da Saúde, Dourados, MS, Brasil.; 2 Universidade Federal de Mato Grosso, Cuiabá, MT, Brasil.; 3 Instituto D’Or de Pesquisa e Ensino - IDOR, São Luís, MA, Brasil.; 4 Universidade Federal do Maranhão, Hospital Universitário - Ebserh, São Luís, MA, Brasil.

**Keywords:** Epidemiology, Fungal Infection, Case reports, Chromoblastomycosis, Chromomycosis

## Abstract

Chromoblastomycosis is a skin infection caused by melanized fungi that primarily affects rural workers. This study aimed to analyze the clinical and epidemiological manifestations of chromoblastomycosis in Brazil through an extensive literature review. A review of case reports or series of cases in English and Portuguese was conducted using the SciELO, LILACS, SCOPUS, PubMed, and Web of Science databases from 1963 to 2022. A total of 46 articles involving 450 patients were identified, among which, 83.1% were male rural workers with a mean age of 52.2 years. The clinical manifestations were most commonly observed in the lower extremities (78.7%). The most frequent clinical presentations of the disease were verrucous lesions and plaques. *Fonsecaea* and *Rhinocladiella* spp. were the most common agents responsible for chromoblastomycosis. Most cured cases were treated with itraconazole, either as monotherapy or in combination with other antifungals, surgery, or cryosurgery. Chromoblastomycosis affects hundreds of rural workers in Brazil, leading to financial disabilities as well as personal and family losses. It is essential to prioritize epidemiological surveillance and ensure the early diagnosis of this disease to reveal its true prevalence, guide resource allocation, improve diagnosis, ensure early treatment, and implement preventive measures.

## INTRODUCTION

Chromoblastomycosis (CBM) is a chronic infection that affects the superficial layers of the skin as well as the deeper tissues. The disease mainly begins with traumatic contact with hyphal fragments in host tissues or the presence of conidia^1^ ^-^ ^9^. This disease mainly affects men of working age, although cases have been reported in very young and elderly patients with a reported age range of 2-99 years^10^. Clinical data on chromoblastomycoses were first reported in Brazil in 1914; however, Terra et al. (1922) first reported the disease ^6^ ^,^ ^11^. Individuals are often in contact with plantations through gardening, farming, logging, and selling raw materials. Therefore, CBM is considered an implantation disease^12^ ^-^ ^17^. The most common etiological agent is *Fonsecaea* spp^18^ ^-^ ^22^. 

Health services in most endemic areas lack professionals trained in the early diagnosis and clinical management of CBM, resulting in a lack of skin biopsies, direct microscopy, histopathology with fungal stains, or fungal culture^22^ ^-^ ^25^. Consequently, patients are usually diagnosed several years after clinical manifestations, increasing the risk of sequelae. The Brazilian Government, through the Ministry of Health, offers itraconazole free of charge for all systemic and implantable mycoses, including CBM. The doses of itraconazole used in CBM therapy range from 200-400 mg/day depending on the severity of the disease. Most patients with mild-to-moderate clinical forms respond to a long-term therapy with a daily dose of 200 mg of itraconazole ^26^ ^-^ ^29^.

Effective treatment and outcomes depend on the causative agent and the length, width, and depth of the CBM lesion. The goal of treatment for individuals with small early lesions is to achieve complete cure. However, for larger lesions, therapy may be long term and ineffective, making it important to focus on controlling the disease and its complications^30^ ^-^ ^35^. We conducted a literature review of case reports to assess the epidemiological and clinical characteristics of CBM in Brazil.

## MATERIALS AND METHODS

The primary question that motivated this review was “What is the current status and detailed characteristics of CBM cases reported in Brazil over the past several decades?” To address this question, a comprehensive literature search was conducted using the following primary databases: SciELO, LILACS, SCOPUS, PubMed, and Web of Science, from January 1963 to December 2022, using the following terms to retrieve the articles: “Chromoblastomycosis and case reports in Brazil,” “Chromoblastomycosis” OR “Cromoblastomicose” OR “Chromomycoses” OR “Cromomicose” AND “Brazil” OR “Brasil.” The review followed the guidelines of the Preferred Reporting Items for Systematic Reviews and Meta-Analyses (PRISMA) statement, which can be accessed at http://www.prisma-statement.org. Only cases reported in English and Portuguese were reviewed, and only papers containing detailed information on individually reported cases were retrieved, reviewed, and subjected to a thorough analysis. Although reports presenting aggregated data are referenced in the Discussion section, they were intentionally excluded from the current review. The references of the retrieved articles were analyzed to identify additional articles that may have been omitted from this search strategy. Cases of localized CBM confirmed by histopathology or KOH mounts were included in this review, regardless of whether there was a confirmed culture. To minimize potential biases in our literature search, we adopted a rigorous method in which two independent reviewers examined the titles and abstracts to select relevant articles based on the predefined inclusion criteria. Discrepancies were resolved by consensus or consultation with a third reviewer when necessary. This process was designed to minimize selection bias and ensure a thorough and unbiased representation of the literature. 

## RESULTS

### ● Study Selection Method

**FIGURE 1:**
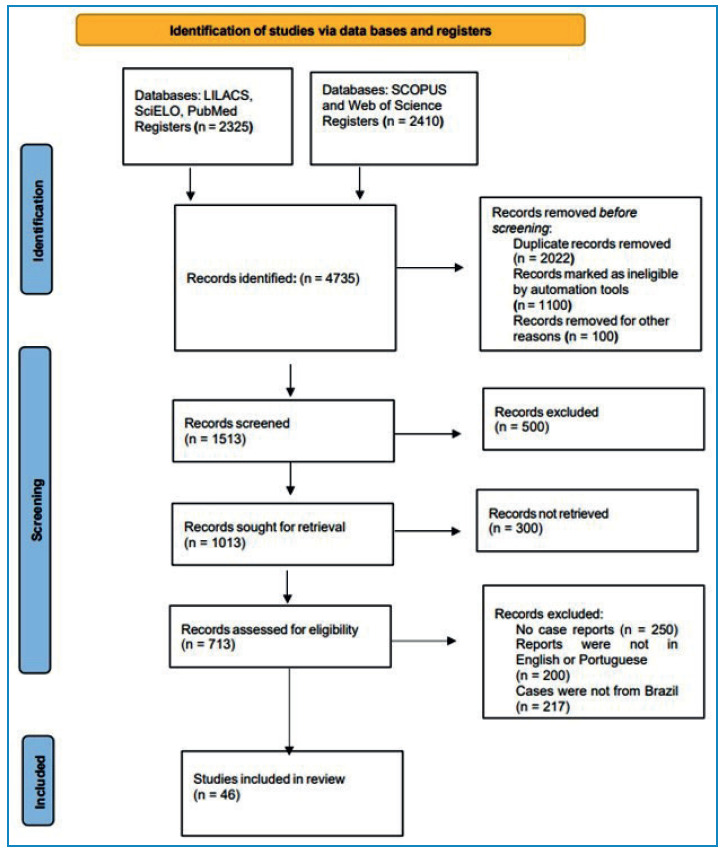
Flowchart depicting the methodology utilized for literature review.

A total of 450 patients with CBM were identified in 46 studies conducted between January 1963 and December 2022. No additional case reports were found in the literature of the selected studies (Figure 1).

### ● Age and sex

The mean patient age found from the studies was 52.2 years (minimum age, 5 years; maximum age, 91 years). Information regarding sex was available for 426 of the 450 patients, of whom 83.1% (374/450) were male, 13.7% (62/450) were female, and 5.7% (26/450) did not indicate their sex. The male: female ratio was 7:1. The mean age at presentation for male and female patients was 56.34 and 46.31 years, respectively (Table 1).

**TABLE 1:** Clinical and demographical differences related to the occurrence of Chromoblastomycosis in different regions of Brazil**.**

Variables	Southeast region 12.2% (n = 55)	South region 31.3% (n = 141)	Northeast region 49.7% (n = 224)	North region 4.2% (n = 18)	Midwest region 2.6% (n = 12)
**Age (mean)**	47.6 (25-83)	53.5 (21-82)	55.8 (15-91)	53.6 (27-77)	50.5 (5-56)
**Sex: Male/Female (n)**	49/4	103/28	194/26	17/3	11/1
**Rural occupation**	17 (5%)	96 (29%)	203 (61.5%)	14 (4.2%)	7 (2.3%)
**History of trauma**	6	20	190	1	1
**Sites of lesions**					
Lower limbs	19	178	169	13	12
Upper limbs	16	44	34	1	0
Face, head, neck	5	20	10	1	0
Trunk	0	3	3	0	0
**Type of lesion**					
Verrucous	20	77	115	14	6
Plaque	10	8	101	8	6
Nodular	2	6	48	7	0
Tumoral	3	3	7	3	0
Ulcer	5	0	21	2	0
Scarring	1	3	20	1	0
**Etiological agent**					
*Fonsecaea* spp.	101	8	300	-	18
*Rhinocladiella* spp.	3	1	0	2	-
*Phialophora* spp*.*	1	4	1	-	-
*Exophiala* spp.	2	1	2	1	-
*Cladophialophora* spp.	1	1	-	-	-

### ● Geographical location

**FIGURE 2:**
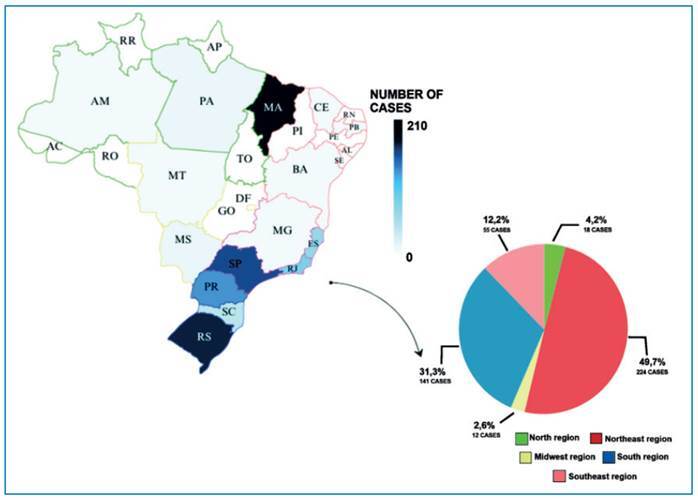
Number of cases of chromoblastomycosis by Brazilian states. The map was drawn based on the case reports available in the literature. The states in red represent the North-east region; those in blue, the South region; those in pink, the South-east region; those in green, the North region; and those in yellow, the Central-west region of Brazil. States of Brazil: **Acre:** AC; **Alagoas:** AL; **Amapá:** AP; **Amazonas:** AM; **Bahia:** BA; **Ceará:** CE; **Espírito Santo:** ES; **Goiás:** GO; **Maranhão:** MA; **Mato Grosso:** MT; **Mato Grosso do Sul:** MS; **Minas Gerais:** MG; **Pará:** PA; **Paraíba:** PB; **Paraná:** PR; **Pernambuco:** PE; **Piauí:** PI; **Rio de Janeiro:** RJ; **Rio Grande do Norte:** RN; **Rio Grande do Sul:** RS; **Rondônia:** RO; **Roraima:** RR; **Santa Catarina:** SC; **São Paulo:** SP; **Sergipe:** SE; **Tocantins:** TO. The graph represents the percentage of cases for the respective regions described on the map.

The largest number of cases was recorded in the north-east region (49.7%, 224/450), followed by the south (31.3%, 141/450), southeast (12.2%, 55/450), north (4.2%, 18/450), and mid-west (2.6 %, 12/450) regions. The distribution of the reported cases by state is shown in Figure 2. 

### ● Occupation

Occupational histories were available for 77.5% (349/450) of the patients. Most (94.55%, 330/349) worked with soil or were directly or indirectly involved with land use-based occupations.

### ● History of trauma

Half of the reported cases (48.5%, 218/450) had a history of trauma, and 51.5% did not remember or denied a history of trauma prior to the onset of the manifestations (51.5%, 232/450).

### ● Site of involvement

Lesions were reported in 94% (423/450) of the patients. The most affected site was the lower limb (78.7% cases, 333/423), followed by the upper limb (22.3% cases, 95/423) followed by the face, head, and neck (8.5%, 36/423). Six percent (27/450) of the patients did not report the affected sites.

### ● Lesion category

**FIGURE 3:**
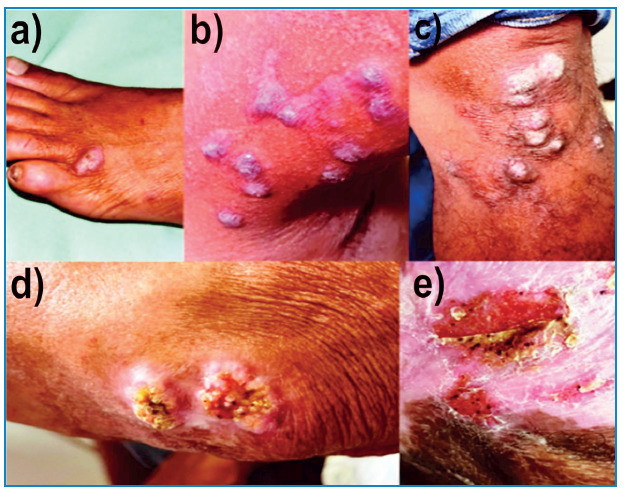
Clinical presentation of Chromoblastomycosis. **a)** Scarring, **b)** Nodular lesion, **c)** Coalescing warty nodules forming plaque, **d)** Verrucous, **e)** Verrucous plaque.

The majority of lesions were verrucous (50.4%, 227/450), followed by plaque (28.4%, 128/450), nodular (14%, 63/450), ulcerative (6.3%, 28/450), scarring (5.6%, 25/450), and tumors (3.6%, 16/450) (Figure 3). More than one type of lesion was observed in 27% of cases (data available for 120/450 cases). The lesions were plaques and verrucous in 41.6% (50/120) of the cases, nodular and verrucous in 33% (40/120), plaques and nodules in 16.6% (20/120), verrucous and ulcerative in 6.6% (8/120), and tumors and verrucous in 1.6% (2/120).

### ● Laboratory Diagnosis

Laboratory diagnoses were performed by histopathology and PCR in 42.4% (191/450), histopathology in 24% (108/450), culture in 20.8% (94/450), PCR in 7.5% (34/450), and culture plus histopathology in 5.1% (23/450) cases.

The majority of the pathogenic species belonged to the genus *Fonsecaea* spp., with *Fonsecaea pedrosoi* being the most common etiological agent (90.8%, 409/450), followed by *F. monophora* 2.6%, 12/450), *F. nubica* (1.1%, 5/450), and *F. pugnacius* (0.2%, 1/450). The second most common pathogenic genus was *Rhinocladiella* spp., with the most commonly found species being *Rhinocladiella aquaspersa* 0.6% (3/450), *R. tropicalis* 0.4% (2/450), and *R. similis* 0.4%(2/450). The third most common pathogenic genus was *Phialophora* spp., with the only species found being *Phialophora verrucosa* (1.3% (6/450). This was followed by *Exophiala* spp., of which the most common etiological agents were *Exophiala jeanselmei* 0.8% (4/450), *E. ludovicensis* (0.2% (1/450), *E. spinifera* (0.2% (1/450), *E. bergeri* (0.2% (1/450), and *Cladophialophora carrioni* 0.4% (2/450). All cases are detailed in the Supplementary Material.

### ● Therapeutic strategy

Information on the treatment modalities was provided for 205 patients. The most commonly prescribed medication, either alone or in combination with other drugs or physical methods, was itraconazole (65.3%, 134/205), followed by itraconazole + cryotherapy (18%, 37/205). Other treatment options, such as surgical removal, were provided to 5.3% (11/205) of patients. The most commonly used combined treatment was itraconazole + cryosurgery, administered in 4.8% (10/205) of cases, followed by itraconazole + surgical resection in 3.9% (08/205), and itraconazole + topical cream in 2.4% (05/205) of the patients.

## DISCUSSION

CBM is one of the most common implantation mycoses found in tropical and subtropical regions worldwide, with sporadic reports in temperate regions^36^ ^-^ ^40^. Although rarely fatal, CBM is a serious disease owing to its high morbidity. The disorder begins with skin-colored papules that gradually develop into nodules or plaques with scaly and verrucous surfaces, eventually adopting a tumoral appearance (similar to a cauliflower), with a verrucous and scarred appearance. In the advanced stages, a single patient may present with more than one type of lesion, which can lead to severe disfigurement and may ultimately require limb amputation. The disease has a low cure rate and high recurrence rate, especially in chronic and extensive cases^41^.

CBM is associated with dematiaceous fungi, and primarily affects individuals living in tropical and temperate regions. Our review revealed that the mean age at diagnosis was 52.2 years, which may be attributed to the long incubation period and late detection of the disease^41^. Our findings are consistent with those from Madagascar, where CBM is less commonly diagnosed among younger individuals^42^. In addition, we observed a predominance of affected men, suggesting that high estrogen levels may protect women from CBM^43^.

Approximately 94.5% of patients diagnosed with CBM reported direct or indirect involvement in agricultural activities. Agricultural work is strongly associated with the pathogenesis of CBM, because the environmental sources of the pathogenic black fungi include soil, vegetables, and wood. This finding is consistent with previous studies reporting that >70% of patients with CBM are farmers^44^ ^-^ ^47^. CBM is a global disease that affects several regions, including Latin America, Africa, South Asia, Australia, and Europe. In Brazil, CBM is present in all states with an estimated prevalence of 1 in 196,000 inhabitants. However, some hyper-endemic regions have a much higher prevalence^48^ ^-^ ^51^. Most cases reviewed in this study were from the north-eastern region, with Maranhão reporting the highest number of reported cases. This area has different tropical climate patterns and previous studies have identified more than 17 environmental reservoirs of the black fungi in the northern mesoregion of Maranhão^52^_._

The primary route of CBM infection is traumatic inoculation, and the distal extremities are vulnerable to infection because of common injury sites. The lower limb was implicated in 78.7% of the cases, likely due to the farmers’ proximity to the soil and lack of protective personal equipment. In contrast, some countries have reported the involvement of the upper limbs in work-related injuries with cacti^53^ ^-^ ^55^. In Brazil, verrucous lesions were the most common type (50.4%), with significant rates of verruciform and plaque-type lesions. Verrucous lesions have a cauliflower-like appearance, are hyperkeratotic, dry, have black dots, and can ulcerate and discharge pus. Advanced cases may develop lymphadenopathy, elephantiasis, and lymphedema^56^.

The diagnosis of CBM requires isolation and identification of the etiological agent, which typically appears as slow-growing, dark-pigmented colonies on routine mycology culture media. Despite their importance, cultures can become contaminated and require molecular identification for species definition. The most sensitive diagnostic methods include direct examination of skin scrapings, pus, or exudates, as well as histopathology. In our review, histopathology combined with molecular diagnostic methods was the most commonly used diagnostic approach (42.4%). Despite the importance of molecular diagnosis for the accurate identification of the etiological agents of CBM, our review found that only a small proportion (7.5%) of cases were diagnosed solely by PCR. This highlights a significant gap in the use of molecular identification methods, which are crucial for confirming the epidemiological aspects of the disease and understanding the true diversity of the causal agents. In low-income countries, the visualization of muriform cells in the tissue debris involved is a common and rapid diagnostic tool for CBM. In Brazil, most cases are caused by *Fonsecaea* spp., particularly *F. pedrosoi*, which is consistent with global reports identifying the species as the primary etiological agent^57^ ^,^ ^58^. However, in some regions, precise identification of causal agents remains challenging. The fragility of the data, especially in cases where molecular identification was not possible, should be acknowledged. Cases in which the diagnosis relies solely on histopathology or clinical presentation may introduce some degree of uncertainty in the epidemiological conclusions. Therefore, future studies should prioritize integrating molecular diagnostics to ensure more accurate identification of the fungal agents involved in CBM, thereby refining the understanding of its epidemiology. 

Amphotericin B is indicated for severe clinical forms that do not respond to itraconazole therapy and is considered an optional treatment. 5-Fluorocytosine (5-FC) should be used in combination with itraconazole rather than as a monotherapy in refractory cases^59^ ^-^ ^60^. The most effective treatment is a considerable dose of itraconazole and terbinafine administered for approximately 1 year. Other treatment modalities such as cryotherapy and local heat are also used to treat small lesions; however, incidences of recurrence have been recorded. The use of itraconazole combined with 5-FC may aid recovery, and the combination of these two medications has been effective against CBM and various forms of subcutaneous mycoses^59^ ^,^ ^61^. The estimated global burden of CBM is 10,000 cases annually, highlighting the importance of understanding the evolution of the disease^62^. It is important to establish an epidemiological surveillance system that includes mandatory case reporting, active case findings, and molecular identification of etiological agents. Furthermore, several publications did not include follow-up records, and there was insufficient information regarding treatment outcomes^63^. Therefore, infection should be prevented to avoid traumatic transcutaneous environmental inoculation in susceptible patients. The information provided may be useful in managing future studies on the epidemiological characteristics of CBM, and in developing plans for diagnosing and recognizing the disease.

## References

[1] Bittencourt AL, Londero AT, Andrade JAF (1994). Cromoblastomicose auricular. Rev Inst Med Trop.

[2] Machado F, Basílio A, Hammerschmidt M, Mukai MM, Werner B, Lameira Pinheiro R (2012). Mucormycosis and chromoblastomycosis occurring in a patient with leprosy type 2 reaction under prolonged corticosteroid and thalidomide therapy. An Bras Dermatol.

[3] Matte SMW, Lopes JO, Melo IS, Espadim LER, Pinto MS (1997). Cromoblastomicose no Rio Grande do Sul. Rev Soc Bras Med Trop.

[4] Ogawa MM, Peternelli MP, Enokihara MMSS, Nishikaku AS, Gonçalves SS, Tomimori J (2016). Spectral Manifestation of Melanized Fungal Infections in Kidney Transplant Recipients: Report of Six Cases. Mycopathologia.

[5] Queiroz-Telles F, Nucci M, Colombo AL, Tobón A, Restrepo A (2011). Mycoses of implantation in Latin America: An overview of epidemiology, clinical manifestations, diagnosis and treatment. Medical Mycology.

[6] Queiroz-Telles F, de Hoog S, Santos DWCL, Salgado CG, Vicente VA, Bonifaz A (2017). Chromoblastomycosis. Clin Microbiol.

[7] De M Aria C, Silva PE, Branco FC, Unda R, Silva R, Costa JML (1994). Relato de caso associação de cromobrastomicose e hanseníase: relato de dois casos. Rev Soc Bras Med Trop.

[8] Queiroz-Telles F, Eillus JN, Botldlgnon GE, Lameira RPBS, Van Cutsem J, Cauwenbergh G (1992). Pharmacology and therapeutics itraconazole in the treatment of chromoblastomycosis due to Fonsecaea pedrosoi. Int J Dermatol.

[9] Zaror L, Fischman O, Pereira CA, Felipe RG, Gregório LC, Castelo A (1987). A Case of Primary Nasal Chromoblastomycosis. Mycosen.

[10] Santos DWCL, C de MP e. S de Azevedo, Vicente VA, Queiroz-Telles F, Rodrigues AM, de Hoog GS (2021). The global burden of chromoblastomycosis. PLoS Negl Trop Dis.

[11] Castro RM, Castro LGM (1987). On the Priority of Description of Chromomycosis. Mykosen.

[12] Diniz YCM, Simões EA, Bomfim MRQ, Conceição PCR, Silva RR, Marques SG (2019). Chromoblastomycosis and Chagas’ disease: a case study in the Brazilian Northeast. Braz. J. of Develop.

[13] Gon AS, Minelli L (2006). Melanonoma in a long-stading lesion of chromoblastomycosis. Int J Dermatol.

[14] Höfling-Lima AL, Guarro J, De Freitas D, Godoy P, Gené J, de Souza LB (2005). Arq Bras Oftalmol.

[15] González GM, Rojas OC, González JG, Kang Y, De Hoog GS (2013). Chromoblastomycosis caused by Rhinocladiella aquaspersa. Med Mycol Case Rep.

[16] Santos DWCL, Vicente VA, Weiss VA, Hoog GS, Gomes RR, Batista EMM (2020). Chromoblastomycosis in an endemic area of brazil: A clinical-epidemiological analysis and a worldwide haplotype network. J Fungi.

[17] Silva CMP, Da Rocha RM, Moreno JS, Dos Remédios M, Branco FC, Silva RR (1995). O babaçu (Orbignya phalerata martins) como provável fator de risco de infecção humana pelo agente da cromoblastomicose no estado do maranhão, brasil. Rev Soc Bras Med Trop.

[18] De Azevedo CMPS, Gomes RR, Vicente VA, Santos DWCL, Marques SG, Do Nascimento MMF (2015). Fonsecaea pugnacius, a novel agent of disseminated chromoblastomycosis. J Clin Microbiol.

[19] Carvalho GSM, Calbucci KBCV, Lellis RF, Veasey JV (2021). Presence of hyphae in chromoblastomycosis examinations: an enigma to be solved. An Bras Dermatol.

[20] Cleinman IB, Gonçalves SS, Nucci M, Quintella DC, Halpern M, Akiti T (2017). Respiratory Tract Infection Caused by Fonsecaea monophora After Kidney Transplantation. Mycopathologia.

[21] Coelho RA, Brito-Santos F, Figueiredo-Carvalho MHG, Silva JVS, Gutierrez-Galhardo MC, do Valle ACF (2018). Molecular identification and antifungal susceptibility profiles of clinical strains of Fonsecaea spp. isolated from patients with chromoblastomycosis in Rio de Janeiro, Brazil. PLoS Negl Trop Dis.

[22] Daboit TC, Duquia RP, Magagnin CM, Mendes SDC, Castrillón MR, Steglich R (2012). A case of Exophiala spinifera infection in Southern Brazil: Molecular identification and antifungal susceptibility. Med Mycol Case Rep.

[23] França K, Villa RT, de Bastos VRA, Almeida ACC, Massucatti K, Fukumaru D (2011). Auricular Chromoblastomycosis: A Case Report and Review of Published Literature. Mycopathologia.

[24] Passero LFD, Cavallone IN, Belda W (2021). Reviewing the etiologic agents, microbe-host relationship, immune response, diagnosis, and treatment in chromoblastomycosis. J Immunol Res.

[25] Veasey JV, Machado B de AR, Lellis RF, Muramatu LH, Zaitz C (2015). Tumoral chromoblastomycosis: A rare manifestation with typical complementary exams. An Bras Dermatol.

[26] Azevedo CMPS, Marques SG, Santos DWCL, Silva RR, Silva NF, Santos DA (2015). Squamous cell carcinoma derived from chronic chromoblastomycosis in Brazil. Clin Infect Dis.

[27] Queiróz AJR, Pereira DF, Antônio JR (2018). Chromoblastomycosis: clinical experience and review of literature. Int J Dermatol.

[28] Criado PR, Careta MF, Valente NYS, Martins JEC, Rivitti EA, Spina R (2011). Extensive long-standing chromomycosis due to Fonsecaea pedrosoi: Three cases with relevant improvement under voriconazole therapy. J Dermatolog Treat.

[29] Abrams JY, Oster ME, Godfred-Cato SE, Bryant B, Datta SD, Campbell AP (2021). Factors linked to severe outcomes in multisystem inflammatory syndrome in children (MIS-C) in the USA: a retrospective surveillance study. Lancet Child Adolesc Health.

[30] Badali H, Bonifaz A, Barrón-Tapia T, Vázquez-González D, Estrada-Aguilar L, Oliveira NMC (2010). Rhinocladiella aquaspersa, proven agent of verrucous skin infection and a novel type of chromoblastomycosis. Med Mycol.

[31] Belda W, Criado PR, Passero LFD (2020). Successful treatment of chromoblastomycosis caused by Fonsecaea pedrosoi using imiquimod. J Dermatol.

[32] Daboit TC, Stopiglia CDO, Antochevis LC, Heidrich D, Magagnin CM, Vettorato G (2011). Sensibilidade a antifúngicos de isolado clínico de *Fonsecaea pedrosoi* oriundo paciente recidivado após tratamento com itraconazaol.

[33] Hoffmann CC, Danucalov IP, Purim KSM, Queiroz-Telles F (2011). Infecções causadas por fungos demácios e suas correlações anátomo-clínicas. An Bras Dermatol.

[34] Zanini M (2012). Tratamento de cromomicose com criocirurgia e itraconazol sistem̂ico. Med Cutan Ibero Lat Am.

[35] De Andrade TS, De Almeida AMZ, Basano SDA, Takagi EH, Szeszs MW, Melhem MSC (2020). Chromoblastomycosis in the Amazon region, Brazil, caused by Fonsecaea pedrosoi, Fonsecaea nubica, and Rhinocladiella similis: Clinicopathology, susceptibility, and molecular identification. Med Mycol.

[36] Belda W, Criado PR, Casteleti P, Passero LFD (2021). Chromoblastomycosis evolving to sarcomatoid squamous cell carcinoma: A case report. Dermatol Reports.

[37] Belda W, Casolato ATS, Luppi JB, Passero LFD (2021). Managing chromoblastomycosis with acitretin plus imiquimod: A case report on the improvement of cutaneous lesions and reduction of the treatment time. J Dermatol.

[38] Melo ED, Morais PM, Fernandes DCL, Rebello PFB (2020). Case for diagnosis. Pruritic erythematosquamous lesion in the auricle. An Bras Dermatol.

[39] Salgado CG, Silva JP, Silva MB, Costa PF, Salgado UI (2005). Cutaneous diffuse chromoblastomycosis. Lancet Infect Dis.

[40] Mouchaloaut MF, Galhardo MCG, Fialho PCM, Coelho JMCO, Oliveira RMZ, Valle ACF (2008). Cladophialophora carrionii: A rare agent of chromoblastomycosis in Rio de Janeiro State, Brazil. Rev Inst Med Trop S Paulo.

[41] Queiroz-Telles F, Esterre P, Perez-Blanco M, Vitale R, Salgado CG, Bonifaz A (2009). Chromoblastomycosis: An overview of clinical manifestations, diagnosis and treatment. Med Mycol.

[42] Sendrasoa FA, Razanakoto NH, Rakotoarisaona M, Onivola R, Rasamoelina T, Ranaivo IM (2020). Clinical aspects of previously treated chromoblastomycosis: A case series from Madagascar. Int J Infect Dis.

[43] Bonifaz A, Carrasco-Gerard E, Saúl A (2001). Chromoblastomycosis: clinical and mycologic experience of 51 cases. Mycoses.

[44] Marques SG, Silva CMP, Saldanha PC, Rezende MA, Vicente VA, Queiroz-Telles F (2006). Isolation of Fonsecaea pedrosoi from the shell of the babassu coconut (Orbignya phalerata Martius) in the Amazon region of Maranhão Brazil. Nihon Ishinkin Gakkai Zasshi.

[45] Minotto R, Bernardi CDV, Mallmann LF, Edelweiss MIA, Scroferneker ML (2001). Chromoblastomycosis: A review of 100 cases in the state of Rio Grande do Sul, Brazil. J Am Acad Dermatol.

[46] De Bona E, Canton LM, Fuentefria AM (2010). Chromoblastomycosis in Santa Catarina state, Brazil. Rev Cubana Med Trop.

[47] Giraldelli GA, Baka JLCS, Orofino-Costa R, Piñeiro-Maceira J, Barcaui E, Barcaui CB (2022). In vivo reflectance confocal microscopy, dermoscopy, high-frequency ultrasonography, and histopathology features in a case of chromoblastomycosis. PLoS Negl Trop Dis.

[48] Gupta AKP, Taborda PR, Sanzovo AD (2002). Alternate week and combination itraconazole and terbinafine therapy for chromoblastomycosis caused by Fonsecaea pedrosoi in Brazil. Med Mycol.

[49] Silva ACCM, S A, Galvão CES, Marques SG, Saldanha ACR, Silva CMP (1992). Cromoblastomicose produzida por Fonsecaea pedrosoi no estado do Maranhão. I-aspectos clínicos, epidemiológicos e evolutivos. Rev Soc Bras Med Trop.

[50] Marques SG, Silva CMP, Resende MA, Silva AAM, Caldas AJM, Costa JML (2008). Detection of delayed hypersensitivity to Fonsecaea pedrosoi metabolic antigen (chromomycin). Nihon Ishinkin Gakkai Zasshi.

[51] de Brito AC, Bittencourt MJS (2018). Chromoblastomycosis: An etiological, epidemiological, clinical, diagnostic, and treatment update. An Bras Dermatol.

[52] Powell RE (1952). A survey of chromoblastomycosis in Queensland. Aust J Dermatol.

[53] Pires CAA, Xavier MB, Quaresma JAS, Macedo GMM, Sousa BRM, Brito AC (2012). Clinical, epidemiological and mycological report on 65 patients from the Eastern Amazon region with chromoblastomycosis. An Bras Dermatolol.

[54] Nóbrega JPS, Rosemberg S, Adami AM, Heins-Vaccari EM, Lacaz CS, Brito T (2003). Fonsecaea pedrosoi cerebral phaeohyphomycosis (“chromoblastomycosis”). First human culture-proven case reported in Brazil. Rev Inst Trop.

[55] Gomes RR, Vicente VA, Azevedo CMPS, Salgado CG, da Silva MB, Queiroz-Telles F (2016). Molecular Epidemiology of Agents of Human Chromoblastomycosis in Brazil with the Description of Two Novel Species. PLoS Negl Trop Dis.

[56] Almeida APM, Gomes NMFG, Almeida LM, Almeida JLM (2014). Cromomicose: relato de caso e revisão da literature. Rev Soc Bras Clin Med.

[57] Criado PR, Cosenza FD, B W, Ferreira PS (2018). Longitudinal melanonychia due to voriconazole therapy during treatment of chromoblastomycosis. Clin Exp Dermatol.

[58] Guevara A, Siqueira NP, Nery AF, Cavalcante LRS, Hagen F, Hahn RC (2021). Chromoblastomycosis in Latin America and the Caribbean: Epidemiology over the past 50 years. Med Mycol.

[59] De Sousa MDGT, Belda W, Spina R, Lota PR, Valente NS, Brown GD (2014). Topical application of imiquimod as a treatment for chromoblastomycosis. Clin Infect Dis.

[60] Antonello VS, Silva MCA, Cambruzzi E, Kliemann DA, Santos BR, Queiroz-Telles F (2010). Treatment of severe chromoblastomycosis with itraconazole and 5-flucytosine association. Rev Inst Med Trop.

[61] Queiroz-Telles F, Fahal AH, Falci DR, Caceres DH, Chiller T, Pasqualotto AC (2017). Neglected endemic mycoses. Lancet Infect Dis.

[62] Bongomin F, Gago S, Oladele RO, Denning DW (2017). Global and Multi-National Prevalence of Fungal Diseases- Estimatite Precision. J Fungi.

[63] Guevara A, Nery AF, Melhem MSC, Bonfietti L, Rodrigues AM, Hagen F (2022). Molecular epidemiology and clinical-laboratory aspects of chromoblastomycosis in Mato Grosso, Brazil. Mycoses.

