# Supplementary material for: Chromoblastomycosis in Brazil: A review of 450 published cases
Source: Rev Soc Bras Med Trop. 2024 Nov 15;57:e00205-2024. doi: 10.1590/0037-8682-0132-2024 (PMC11654747; doi:10.1590/0037-8682-0132-2024)
Supplement: Supplementary file 1 [file 1678-9849-rsbmt-57-e00205-2024-supp1.pdf]

Supplementary Material 01. List of case reports of Chromoblastomycosis in Brazil.

Case	Reference	Year	Age/ Sex	Rural occupation	Brazilian State	Clinical presentation	Diagnosis	Spécies	Treatment	Outcome
1.* (100 cases)	[1]	1963-1998	78/H 22/ M	Yes (79% ofcases)	Rio Grandedo Sul	Verrucous (53% of cases)	Histopathological	*Fonsecaea pedrosoi* (96 cases) *Phialophora verrucosa* (4 cases)	Anfotericina B, 5-fluorocytosine, itraconazole, methotrexate cetoconazole, radiotherapy, criocirugy	NA
2.* (191 cases)	[2]	1989-2018	148/ H 43/ M	Yes (85% ofcases)	Maranhão	Verrucous (55% of cases)	Molecular identification + Histopathological	*Fonsecaea pedrosoi* (186 cases) *F. monophora* (1 case) *F. pugnacius* (1 case)	Itraconazole, cryoteraphy	not cured

								Rhinocladiella tropicalis (2 cases) *Cyphellophora ludovicensis* (1 case)		
3.	[3]	1987	44/F	no	São Paulo	Nodular	Culture	*Fonsecaea pedrosoi*	NA	NA
4.	[4]	1990	48/ M	yes	Espirito Santo	Verrucous and ulcer	Culture	*Fonsecaea pedrosoi*	5-fluorocytosine, thiabendazole and ketoconazole	NA
5.	[4]	1990	83/ M	yes	Espirito Santo	Verrucous and ulcer	Culture	*Fonsecaea pedrosoi*	5-fluorocytosine, thiabendazole and ketoconazole	NA

4.	[4]	1990	55/ M	yes	Espirito Santo	Verrucous	Culture	*Phialophora verrucosa*	5-fluorocytosine, thiabendazole and ketoconazole	NA
5.	[4]	1990	70/ M	yes	Espirito Santo	Verrucous	Culture	*Fonsecaea pedrosoi*	5-fluorocytosine, thiabendazole and ketoconazole	NA
6.	[4]	1990	75/ M	yes	Espirito Santo	Verrucous and ulcer	Culture	*Fonsecaea pedrosoi*	5-fluorocytosine, thiabendazole and ketoconazole	NA
7.	[4]	1990	25/ M	NA	Espirito Santo	Verrucous	Culture	*Fonsecaea spp*	5-fluorocytosine, thiabendazole and ketoconazole	NA

8.	[4]	1990	54/ M	yes	Espirito Santo	NA	Culture	*Fonsecaea pedrosoi*	5-fluorocytosine, thiabendazole and ketoconazole	NA
9.	[4]	1990	39/ M	yes	Espirito Santo	Ulcer	Culture	*Fonsecaea pedrosoi*	5-fluorocytosine, thiabendazole and ketoconazole	NA
10.	[4]	1990	40/ M	yes	Espirito Santo	Verrucous and ulcer	Culture	*Fonsecaea pedrosoi*	5-fluorocytosine, thiabendazole and ketoconazole	NA
11.	[5]	1992	59/ M	yes	Maranhão	NA	Culture	*Fonsecaea pedrosoi*	5- fluorcytosine	NA
12.	[5]	1992	54/F	yes	Maranhão	NA	Culture	*Fonsecaea pedrosoi*	5- fluorcytosine	NA
13.	[5]	1992	48/ M	yes	Ceará	NA	Culture	*Fonsecaea pedrosoi*	5- fluorcytosine	NA

14.	[5]	1992	51/ M	yes	Maranhão	NA	Culture	*Fonsecaea pedrosoi*	5- fluorcytosine	NA
15.	[5]	1992	39/ M	yes	Maranhão	NA	Culture	*Fonsecaea pedrosoi*	5- fluorcytosine	NA
16.	[5]	1992	67/ M	yes	Maranhão	NA	Culture	*Fonsecaea pedrosoi*	5- fluorcytosine	NA
17.	[5]	1992	51/ M	yes	Maranhão	NA	Culture	*Fonsecaea pedrosoi*	5- fluorcytosine	NA
18.	[5]	1992	65/ M	yes	Maranhão	NA	Culture	*Fonsecaea pedrosoi*	5- fluorcytosine	NA
19.	[5]	1992	57/ M	yes	Maranhão	NA	Culture	*Fonsecaea pedrosoi*	5- fluorcytosine	NA

20.	[5]	1992	37/ M	yes	Maranhão	NA	Culture	*Fonsecaea pedrosoi*	5- fluorcytosine	NA
21.	[5]	1992	55/ M	yes	Maranhão	NA	Culture	*Fonsecaea pedrosoi*	5- fluorcytosine	NA
22.	[5]	1992	51/ M	yes	Maranhão	NA	Culture	*Fonsecaea pedrosoi*	5- fluorcytosine	NA
23.	[5]	1992	39/F	yes	Maranhão	NA	Culture	*Fonsecaea pedrosoi*	5- fluorcytosine	NA
24.	[6]	1992	61/ M	NA	Paraná	Nodular and tumorous	Culture + histopathological	*Fonsecaea pedrosoi*	Amphotericin-B and 5-fluorocytosine cryotherapy	Cured
25.	[6]	1992	56/ M	NA	Paraná	Nodular and verrucous	Culture + histopathological	*Fonsecaea pedrosoi*	Amphotericin-B and 5-fluorocytosine cryotherapy	Cured

26.	[6]	1992	54/F	NA	Paraná	Nodular and tumorous	Culture + histopathological	*Fonsecaea pedrosoi*	Amphotericin-B and 5-fluorocytosine cryotherapy	Cured
27.	[6]	1992	81/M	NA	Paraná	Verrucous	Culture + histopathological	*Fonsecaea pedrosoi*	Itraconazole E, amphotericin-B, 5-fluorocytosine+cryotherapy	Cured
28.	[6]	1992	58/M	NA	Paraná	Verrucous and scarring	Culture + histopathological	*Fonsecaea pedrosoi*	Itraconazole E, amphotericin-B, 5-fluorocytosine+cryotherapy	Cured
29.	[6]	1992	50/M	NA	Paraná	Plaque	Culture + histopathological	*Fonsecaea pedrosoi*	Itraconazole E, amphotericin-B, 5-fluorocytosine+cryotherapy	Cured

30.	[6]	1992	61/ M	NA	Paraná	Verrucous and scarring	Culture + histopathological	*Fonsecaea pedrosoi*	Itraconazole E, amphotericin-B, 5-fluorocytosine+cryotherapy	Cured
31.	[6]	1992	75/ M	NA	Paraná	Nodular and tumorous	Culture + histopathological	*Fonsecaea pedrosoi*	Itraconazole E, amphotericin-B, 5-fluorocytosine+cryotherapy	cured
32.	[6]	1992	66/ M	NA	Paraná	Verrucous and plaque	Culture + histopathological	*Fonsecaea pedrosoi*	Itraconazole E, amphotericin-B, 5-fluorocytosine+cryotherapy	cured
33.	[6]	1992	65/ M	NA	Paraná	Nodular and tumorous	Culture + histopathological	*Fonsecaea pedrosoi*	Itraconazole E, amphotericin-B, 5-fluorocytosine+cryotherapy	cured

34.	[6]	1992	72/ M	NA	Paraná	Verrucous	Culture + histopathological	*Fonsecaea pedrosoi*	Itraconazole E, amphotericin-B, 5-fluorocytosine+cryotherapy	cured
35.	[6]	1992	33/F	NA	Paraná	nodular and verrucous	Culture + histopathological	*Fonsecaea pedrosoi*	Itraconazole E, amphotericin-B, 5-fluorocytosine+cryotherapy	cured
36.	[6]	1992	66/ M	NA	Paraná	plaque	Culture + histopathological	*Fonsecaea pedrosoi*	Itraconazole E, amphotericin-B, 5-fluorocytosine+cryotherapy	cured
37.	[6]	1992	82/ M	NA	Paraná	Verrucous and plaque	Culture + histopathological	*Fonsecaea pedrosoi*	Itraconazole E, amphotericin-B, 5-fluorocytosine+cryotherapy	cured

38.	[6]	1992	46/ M	NA	Paraná	verrucous	Culture + histopathological	*Fonsecaea pedrosoi*	Itraconazole E, amphotericin-B, 5-fluorocytosine+cryotherapy	cured
39.	[6]	1992	38/F	NA	Paraná	verrucous and plaque	Culture + histopathological	*Fonsecaea pedrosoi*	Itraconazole E, amphotericin-B, 5-fluorocytosine+cryotherapy	cured
40.	[6]	1992	42/ M	NA	Paraná	verrucous	Culture + histopathological	*Fonsecaea pedrosoi*	Itraconazole E, amphotericin-B, 5-fluorocytosine+cryotherapy	cured
41.	[6]	1992	53/ M	NA	Paraná	verrucous	Culture + histopathological	*Fonsecaea pedrosoi*	Itraconazole E, amphotericin-B, 5-fluorocytosine+cryotherapy	cured

42.	[6]	1992	46/M	NA	Paraná	verrucous	Culture + histopathological	*Fonsecaea pedrosoi*	Itraconazole E, amphotericin-B, 5-fluorocytosine+cryotherapy	Cured
43.	[7]	1994	67/M	yes	Maranhão	Verrucous	Culture + histopathological	*Fonsecaea pedrosoi*	NA	NA
44.	[7]	1994	50/M	yes	Maranhão	Verrucous	Culture + histopathological	*Fonsecaea pedrosoi*	NA	NA
45.	[8]	1994	66/M	no	Bahia	Ulcer	Culture + histopathological	*Phialophora verrucosa*	NA	NA
46.	[9]	1995	51/M	yes	Maranhão	Verrucous	Culture + histopathological	*Fonsecaea pedrosoi*	NA	Survived
47.	[9]	1995	57/F	yes	Maranhão	Wart vegetative	Culture + histopathological	*Fonsecaea pedrosoi*	NA	NA

48.	[10]	1997	NA	yes	Rio Grande do Sul	Nodular	Culture	*Fonsecaea pedrosoi*	Itraconazole+surgical resection and local heat	Survived
49.	[10]	1997	NA	yes	Rio Grande do Sul	Verrucous	Culture	*Fonsecaea pedrosoi*	Itraconazole+ surgical resection	Survived
50.	[10]	1997	NA	yes	Rio Grande do Sul	Verrucous	Culture	*Fonsecaea pedrosoi*	5-fluorocytosine+ surgical resection	Survived
51.	[10]	1997	NA	yes	Rio Grande do Sul	Verrucous	Culture	*Fonsecaea pedrosoi*	Amphotericin-B, 5-fluorocytosine+surgic al resection+local heat	NA
52.	[10]	1997	NA	yes	Rio Grande do Sul	Verrucous	Culture	*Fonsecaea pedrosoi*	Amphotericin-B, 5-fluorocytosine+surgic al resection+local heat	NA

53.	[10]	1997	NA	yes	Rio Grande do Sul	Verrucous	Culture	*Fonsecaea pedrosoi*	NA	NA
54.	[10]	1997	NA	yes	Rio Grande do Sul	Verrucous	Culture	*Fonsecaea pedrosoi*	Anfotercina-B, itraconazole + Local heat+electrocoagulation	NA
55.	[10]	1997	NA	yes	Rio Grande do Sul	Verrucous	Culture	*Fonsecaea pedrosoi*	NA	NA
56.	[10]	1997	NA	yes	Rio Grande do Sul	Verrucous	Culture	*Fonsecaea pedrosoi*	Itraconazole+ local heat	NA
57.	[10]	1997	NA	yes	Rio Grande do Sul	Verrucous	Culture	*Fonsecaea pedrosoi*	Surgical resection+local heat	NA
58.	[10]	1997	NA	yes	Rio Grande do Sul	Verrucous	Culture	*Fonsecaea pedrosoi*	Surgical resection	NA

59.	[10]	1997	NA	yes	Rio Grande do Sul	Verrucous	Culture	*Fonsecaea pedrosoi*	Itraconazole+ surgical resection	Survived
60.*	[11] (12 cases)	2002-2003	NA	yes	Maranhão	Verrucous	Culture	*Fonsecaea pedrosoi*	Itraconazole	Survived
60.	[12]	2002	61/ M	NA	São Paulo	NA	Culture	*Fonsecaea pedrosoi*	Ketoconazole, terbinafine+cryotherapy	Survived
61.	[12]	2002	50/ M	NA	São Paulo	Verrucous	Culture	*Fonsecaea pedrosoi*	Itraconazole, terbinafine	Survived
62.	[12]	2002	55/F	NA	São Paulo	Scarring	Culture	*Fonsecaea pedrosoi*	Ketoconazole, terbinafine+cryotherapy	Survived

63.	[12]	2002	39/ M	NA	São Paulo	Verrucous	Culture	*Fonsecaea pedrosoi*	Itraconazole, terbinafine	Survived
64.	[13]	2003	28/ M	yes	São Paulo	Tumorous	Culture	*Fonsecaea pedrosoi*	Itraconazole	Survived
65.	[14]	2004	52/ M	yes	Maranhão	Plaque	Culture	*Rhinocladiella aquaspersa*	Cetoconazole	NA
66.	[15]	2005	18/ M	NA	São Paulo	Ulcer	Culture	*Fonsecaea pedrosoi*	Natamycin, ketoconazole, amphotericin-B	NA
67.	[16]	2005	43/ M	no	Pará	Nodular	Culture	*Fonsecaea pedrosoi*	Dapsone	Died
68.	[17]	2006	70/ M	yes	Paraná	Verrucous and scarring	Histopathological	*Fonsecaea pedrosoi.*	NA	Survived

69.	[18]	2008	73/ M	yes	Rio de Janeiro	Tumorous	Culture	*Cladophialophora carrioni*	Itraconazole	NA
70.	[19]	2010	73/ M	yes	Santa Catarina	Plaque	Culture	*Cladophialophora carrioni*	NA	NA
71.	[20]	2010	62/F	yes	Paraíba	Verrucous	Histopathological	*Rhinocladiella aquaspersa*	Itraconazole	Survived
72.	[21]	2011	77/ M	yes	Rondônia	Nodular, tumorous, verrucous, scarring and plaque	Culture	*Fonsecaea pedrosoi*	Intraconazole, terbinafine, cryotherapy with nitrogen	NA
73.	[21]	2011	59/ M	yes	Rondônia	Verrucous	Culture	*Fonsecaea pedrosoi*	Itraconazole, terbinafine, cryotherapy with nitrogen	NA

74.	[21]	2011	44/ M	NA	Rondônia	Verrucous	Culture	*Fonsecaea pedrosoi*	Amphotericin-B+itraconazole, voriconazole	NA
75.	[22]	2011	67/ M	yes	São Paulo	Plaque and nodular	Culture	*Fonsecaea pedrosoi*	Itraconazole, cryotherapy	Survived
76.	[23]	2011	36/ M	NA	Paraná	Nodular	Culture	*Exophiala jeanselmei*	prednisone+cyclosporine+azathioprine	NA
77.	[23]	2011	38/ M	NA	Paraná	Plaque and scarring	Culture	*Exophiala jeanselmei*	Amphotericin B, itraconazole	NA
78.	[23]	2011	59/ M	NA	Paraná	Plaque and scarring	Histopathological	*Exophiala jeanselmei*	Itraconazole	NA
79.	[23]	2011	49/ M	yes	Paraná	NA	Culture	*Exophiala jeanselmei*	Itraconazole sulfonamides + penicilin +	NA

									ketoconazole, amphotericin B	
80.	[24]	2012	28/ M	no	Paraná	NA	Culture	*Fonsecaea pedrosoi*	Amphotericin B	cured
81.	[25]	2012	80/ M	yes	Rio Grande do Sul	NA	Molecular identification	*Exophiala spinifera*	Itraconazole	cured
82.	[26]	2012	59/ M	no	Santa Catarina	Tumorous and verrucous	Histopathological	*Fonsecaea pedrosoi*	Itraconazole	NA
83.	[27]	2013	69/ M	NA	Santa Catarina	NA	Culture	*Fonsecaea pedrosoi.*	Itraconazole +voriconazole + terbinafine +ambphotericin B	NA
84.	[28]	2014	71/ M	no	São Paulo	Verrucous	Culture	*Fonsecaea pedrosoi*	Imiquimod + itraconazole	NA

85.	[28]	2014	70/ M	no	São Paulo	Verrucous	Culture	*Fonsecaea pedrosoi*	Imiquimod + itraconazole, terbinafine	NA
86.	[28]	2014	46/ M	no	São Paulo	Plaque and verrucous	Culture	*Fonsecaea pedrosoi*	Imiquimod + itraconazole + topic	NA
87.	[28]	2014	71/ M	no	São Paulo	Verrucous	Culture	*Fonsecaea pedrosoi*	Imiquimod, itraconazole + terbinafine	NA
88.	[29]	2014	83/ M	Yes	Rio de Janeiro	Verrucous	Culture	*Fonsecaea pedrosoi*	Itraconazole + cryotherapy	cured
89.	[30]	2015	52/ M	yes	Maranhão	Plaque and verrucous	Culture	*Fonsecaea pugnacius*	Amphotericin B + itraconazole, varociconazole	died

90.	[31]	2015	55/ M	NA	Maranhão	Plaque and verrucous	Culture	*Fonsecaea pedrosoi*	Itraconazole, surgical resection	Survived
91.	[31]	2015	81/ M	NA	Maranhão	Verrucous	Culture	*Fonsecaea pedrosoi*	Itraconazole, surgical resection	Survived
92.	[31]	2015	65/ M	NA	Maranhão	Tumorous	Culture	*Fonsecaea pedrosoi*	Itraconazole	Died
93.	[31]	2015	64/ M	NA	Maranhão	Tumorous	Culture	*Fonsecaea pedrosoi*	Itraconazole	Survived
94.	[31]	2015	45/ M	NA	Maranhão	Plaque and verrucous	Culture	*Fonsecaea pedrosoi*	Itraconazole	Survived
95.	[31]	2015	68/ M	NA	Maranhão	Scarring	Culture	*Fonsecaea pedrosoi*	Itraconazole	Survived

96.	[31]	2015	88/ M	NA	Maranhão	Plaque and verrucous	Culture	*Fonsecaea pedrosoi*	Itraconazole, surgical resection	Survived
97.	[32]	2015	77/ M	NA	São Paulo	Tumorous	Culture	*Fonsecaea pedrosoi*	NA	NA
98.	[33]	2016	57/ M	no	Maranhão	Plaque and nodular	Culture	*Fonsecaea pedrosoi.*	Itraconazole, cryosurgery	NA
99.	[33]	2016	65/ M	yes	Maranhão	Plaque and nodular	Culture	*Fonsecaea pedrosoi*	Itraconazole, nitrogen, cryosurgery	Survived
100.	[33]	2016	78/ M	yes	Maranhão	Plaque	Culture	*Fonsecaea pedrosoi*	Itraconazole + terbinafine	Survived
101.	[34]	2016	43/ M	no	São Paulo	Verrucous	Molecular identification	*Fonsecaea monophora*	Tacrolimus+azathioprine+prednisone	Survived

102.	[34]	2016	57/M	no	São Paulo	Plaque	Culture	*Fonsecaea pedrosoi*	Azathioprine+tacrolimus+prednisone, terbinafine	Survived
103.	[34]	2016	60/M	no	São Paulo	NA	Culture	*Fonsecaea pedrosoi*	Tacrolimus+sodium mycophenolate + prednisone, itraconazole	Survived
104.	[34]	2016	54/M	no	São Paulo	Ulcer, nodules and verrucous	Molecular identification	*Exophiala bergeri*	Itraconazole	Survived
105.	[35]	2017	57/F	no	Rio de Janeiro	Plaque	Culture	*Fonsecaea monophora*	Itraconazole	Survived
106.	[36]	2017	52/M	NA	São Paulo	Verrucous	Culture	*Fonsecaea pedrosoi*	Other therapies, voriconazole, cryotherapy	NA

107.	[37]	2018	NA	NA	Rio de Janeiro	NA	Molecular identification	*Fonsecaea pedrosoi.*	NA	NA
108.	[37]	2018	NA	NA	Rio de Janeiro	NA	Molecular identification	*Fonsecaea nubica*	NA	NA
109.	[37]	2018	42/F	no	Rio de Janeiro	NA	Molecular identification	*Fonsecaea pedrosoi.*	Itraconazole+fluconazole+terbinafine, cryosurgery	NA
110.	[37]	2018	72/M	no	Espírito Santo	NA	Molecular identification	*Fonsecaea monophora*	Itraconazole+fluconazole	cured
111.	[37]	2018	65/M	yes	Rio de Janeiro	NA	Molecular identification	*Fonsecaea monophora*	Itraconazole	cured
112.	[37]	2018	50/F	no	Rio de Janeiro	NA	Molecular identification	*Fonsecaea nubica*	Cryosurgery	cured

113.	[37]	2018	36/F	no	Rio de Janeiro	NA	Molecular identification	*Fonsecaea monophora*	Cryosurgery	cured
114..	[37]	2018	45/ M	no	Rio de Janeiro	NA	Molecular identification	*Fonsecaea monophora*	Itraconazole, cryosurgery	cured
115.	[37]	2018	83/ M	no	Rio de Janeiro	NA	Molecular identification	*Fonsecaea nubica*	Itraconazole, cryosurgery	NA
116.	[37]	2018	NA/ M	no	Rio de Janeiro	NA	Molecular identification	*Fonsecaea monophora*	Itraconazole, cryosurgery	NA
117.	[37]	2018	53/ M	no	Rio de Janeiro	NA	Molecular identification	*Fonsecaea monophora*	NA	cured
118.	[37]	2018	35/ M	yes	Rio de Janeiro	NA	Molecular identification	*Fonsecaea monophora*	Cryosurgery	cured

119.	[37]	2018	NA/ M	no	Rio de Janeiro	NA	Molecular identification	*Fonsecaea pedrosoi*	Itraconazole	NA
120.	[37]	2018	NA/ M	no	Rio de Janeiro	NA	Molecular identification	*Fonsecaea pedrosoi*	NA	NA
121.	[37]	2018	60/ M	no	Rio de Janeiro	NA	Molecular identification	*Fonsecaea monophora.*	NA	cured
122.	[37]	2018	58/ M	no	Rio de Janeiro	NA	Molecular identification	*Fonsecaea monophora*	Itraconazole	cured
123.	[37]	2018	65/ M	no	Rio de Janeiro	NA	Molecular identification	*Fonsecaea rubica*	Itraconazole+terbinafine, cryosurgery	cured
124.	[38]	2018	66/ M	yes	São Paulo	Plaque	Culture	*Fonsecaea pedrosoi*	Itraconazole, cryosurgery	Survived

125.	[38]	2018	72/ M	yes	São Paulo	Plaque	Culture	*Fonsecaea pedrosoi*	Itraconazole, cryosurgery	Survived
126.	[38]	2018	54/ M	no	São Paulo	Plaque	Culture	*Fonsecaea pedrosoi*	Cryosurgery	Survived
127.	[38]	2018	63/ M	no	São Paulo	Plaque	Culture	*Fonsecaea pedrosoi*	Itraconazole, cryosurgery	survived
128.	[38]	2018	73/ M	no	São Paulo	Verrucous	Culture	*Fonsecaea pedrosoi*	Itraconazole, shaving+electrocauteri zation	NA
129.	[39]	2019	75/ M	yes	Rondônia	Nodular, tumorous	Molecular identification	*Fonsecaea pedrosoi*	Itraconazole	cured

130.	[39]	2019	27/F	yes	Rondônia	Plaque, verrucous and ulcer	Molecular identification	*Fonsecaea pedrosoi*	Itraconazole, cryotherapy	not cured
131.	[39]	2019	59/M	yes	Rondônia	Plaque, verrucous, ulcer and nodular	Molecular identification	*Fonsecaea nubica*	Itraconazole, cryotherapy	not cured
132.	[39]	2019	66/M	yes	Rondônia	nodular, tumorous	Molecular identification	*Rhinocladiela similis*	Itraconazole, cryotherapy	cured
133.	[39]	2019	57/F	yes	Rondônia	Plaque, verrucous	Molecular identification	*Fonsecaea pedrosoi*	Itraconazole	not cured
134.	[39]	2019	44/M	yes	Rondônia	Plaque, verrucous	Molecular identification	*Fonsecaea pedrosoi*	Itraconazole	not cured

135.	[39]	2019	68/ M	yes	Rondô nia	Plaque, verrucous	Molecular identification	*Rhinocladiela similis*	Itraconazole	not cured
136.	[39]	2019	35/ M	yes	Rondô nia	Plaque, verrucous	Molecular identification	*Fonsecaea pedrosoi*	Itraconazole	not cured
137.	[39]	2019	70/ M	yes	Rondô nia	Plaque, verrucous	Molecular identification	*Fonsecaea pedrosoi*	Itraconazole	not cured
138.	[39]	2019	36/F	no	Rondô nia	Plaque, verrucous	Molecular identification	*Fonsecaea nubica*	Itraconazole	NA
139.	[39]	2019	42/ M	yes	Rondô nia	Plaque, verrucous	Molecular identification	*Fonsecaea pedrosoi*	Itraconazole	NA
140.	[39]	2019	56/ M	yes	Rondô nia	Plaque, verrucous	Molecular identification	*Fonsecaea pedrosoi*	Itraconazole	NA

141.	[40]	2019	56/ M	yes	Maran hão	Plaque and verrucous	Culture	*Fonsecaea pedrosoi*	Itraconazole	NA
142.	[41]	2020	55/ M	no	São Paulo	Plaque and verrucous	Culture	*Fonsecaea pedrosoi*	Itraconazole + fluconazole + ketoconazole + terbinafine + phototherapy, cryotherapy	survived
143.	[42]	2020	57/ M	yes	Amazo nas	Infiltrated	Molecular identification	*Rhinocladiella aquaspersa*	Itraconazole	NA
144.	[43]	2021	45/ M	no	Mato Grosso do Sul	Verrucous	Culture	*Fonsecaea pedrosoi*	Itraconazole + topical imiquimod	survived
145.	[43]	2021	43/ M	yes	São Paulo	Verrucous	Culture	*Fonsecaea pedrosoi*	Topic + imiquimod	survived

146.	[43]	2021	39/ M	no	São Paulo	Verrucous	Culture	*Fonsecaea pedrosoi*	Itraconazole+terbinafine, imiquimod	survived
147.	[44]	2021	56/ M	no	Mato Grosso do Sul	Plaque	Culture	*Fonsecaea pedrosoi*	Itraconazole+neomycin+ oral cephalosporin + itretin, actretin + topical imiquimod	survived
148.	[45]	2021	58/ M	no	Pará	Nodular	Culture	*Fonsecaea pedrosoi*	Itraconazole+surgical excison, cryotherapy	survived
149.	[46]	2022	60/ M	NA	Rio de Janeiro	Verrucous + ulcer	Culture	*Fonsecaea pedrosoi*	NA	NA

NA: Not Available

*They are series of cases that could not be individualized.

References:

1. Minotto R, Bernardi CDV, Mallmann LF, Edelweiss MIA, Scroferneker ML. Chromoblastomycosis: A review of 100 cases in the state ofRio Grande do Sul, Brazil. J Am Acad Dermatol. 2001;44(4):585–92.
2. Santos DWCL, Vicente AV, Weiss GSH, Gomes RR, Batista EMM, Marques SG. Chromoblastomycosis in an Endemic Area of Brazil:A Clinical-Epidemiological Analysis and a Worldwide Haplotype Network. 2020;6(4):204
3. Zaror L, Fischman O, Pereira CA, Felipe RG, Gregório LC, Castelo A. A Case of Primary Nasal Chromoblastomycosis. Mycosen.1987;30(10):468–71.
4. Mattêde MGS, Júnior LP, Coelho CC, Mattêde AF. Dermatite verrucosa cromoparasitária. 1990.;65(2):70-74
5. Silva ACCM, Neto AS, Galvão CES, Marques SG, Saldanha ACR, Silva CMP, et al. Cromoblastomicose produzida por *Fonsecaeapedrosoi* no estado do Maranhão. I- aspectos clínicos, epidemiológicos e evolutivos. Rev Soc Bras Med Trop. 1992;25(1):37-4.
6. Queiroz-Telles F, Eillus JN, Botldlgnon GE, Lameira RPBS, Van Cutsem J, Cauwenbergh G. Pharmacology and therapeutics itraconazole in the treatment of chromoblastomycosis due to *Fonsecaea pedrosoi*. Int J Dermatol. 1992;31(11):805-12.
7. De M Aria C, Silva PE, Branco FC, Unda R, Silva R, Costa JML. Relato de caso associação de cromobrastomicose e hanseníase: relato dedois casos. Rev Soc Bras Med Trop. 1994;27(4):241-44.
8. Bittencourt AL, Londero AT, Andrade JAF. Cromoblastomicose auricular. Rev Inst Med Trop. 1994;36(4):381-83.
9. Silva CMP, Da Rocha RM, Moreno JS, Dos Remédios M, Branco FC, Silva RR, et al. O babaçu (Orbignya phalerata martins) como provável fator de risco de infecção humana pelo agente da cromoblastomicose no estado do maranhão, brasil. Rev Soc Bras Med Trop. 1995;28(1):49-2.
10. Matte SMW, Lopes JO, Melo IS, Espadim LER, Pinto MS. Cromoblastomicose no Rio Grande do Sul. Soc Bras Med Trop. 1997;30(4):309-11.
11. Marques SG, Silva CMP, Resende MA, Silva AAM, Caldas AJM, Costa JML. Detection of delayed hypersensitivity to *Fonsecaea pedrosoi* metabolic antigen (chromomycin). Nihon Ishinkin Gakkai Zasshi. 2008;49(2):95-101.
12. Gupta AKP, Taborda PR, Sanzovo AD. Alternate week and combination itraconazole and terbinafine therapy for chromoblastomycosiscaused by *Fonsecaea pedrosoi* in Brazil. Med Mycol. 2002;40(5):529-34.
13. Nóbrega JPS, Rosemberg S, Adami AM, Heins-Vaccari EM, Lacaz CS, Brito T. *Fonsecaea pedrosoi* cerebral phaeohyphomycosis(”chromoblastomycosis”). First human culture-proven case reported in Brazil. Rev Inst Trop. 2003;45(4):217-20.
14. Marques SG, Pedrozo SCM, Resende MA, Andreata LS, Costa ML. Chromoblastomycosis caused by Rhinocladiella aquaspersa. MedicalMycology 2004; 42(Suppl. 3): 261–5.
15. Höfling-Lima AL, Guarro J, De Freitas D, Godoy P, Gené J, de Souza LB, et al. Clinical treatment of corneal infection due to *Fonsecaeapedrosoi* – Case report. 2005;68(2):270-2.
16. Salgado CG, Silva JP, Silva MB, Costa PF, Salgado UI. Cutaneous diffuse chromoblastomycosis. Lancet Infect Dis. 2005;5(8):528.
17. Gon AS, Minelli L. Melanonoma in a long-stading lesion of chromoblastomycosis. Int J Dermatol. 2006;45(11):1331-3.

18. Mouchalouat MDF, Galhardo MCG, Fialho PCM, Coelho JMCDO, Zancopé-Oliveira RM, Valle ACF. Cladophialophora carrionii: A rareagent of chromoblastomycosis in Rio de Janeiro State, Brazil. Revista Do Instituto de Medicina Tropical de Sao Paulo 2008; 50 (Suppl. 6): 351–3.
19. De Bona E, Canton LM, Fuentefria AM. Chromoblastomycosis in Santa Catarina state, Brazil. Rev Cubana Med Trop. 2010;62(3):254-6.
20. Badali H, Bonifaz A, Barrón-Tapia T, Vázquez-González D, Estrada-Aguilar L, Oliveira NMC, et al. *Rhinocladiella aquaspersa*, proven agent of verrucous skin infection and a novel type of chromoblastomycosis. Med Mycol. 2010;48(5):696–3.
21. Criado PR, Careta MF, Valente NYS, Martins JEC, Rivitti EA, Spina R, et al. Extensive long-standing chromomycosis due to *Fonsecaea pedrosoi*: Three cases with relevant improvement under voriconazole therapy. J Dermatolog Treat. 2011;22(3):167–74.
22. França K, Villa RT, de Bastos VRA, Almeida ACC, Massucatti K, Fukumaru D, et al. Auricular Chromoblastomycosis: A Case Report and Review of Published Literature. Mycopathologia. 2011;172(1):69–2.
23. Hoffmann CC, Danucalov IP, Purim KSM, Queiroz-Telles F. Infecções causadas por fungos demácios e suas correlações anátomo-clinicas.An Bras Dermatol. 2011;86(1):138-41.
24. Machado F, Basílio A, Hammerschmidt M, Mukai MM, Werner B, Lameira Pinheiro R, et al. Mucormycosis and chromoblastomycosis occurring in a patient with leprosy type 2 reaction under prolonged corticosteroid and thalidomide therapy. An Bras Dermatol. 2012;87(5):767-71.
25. Daboit TC, Duquia RP, Magagnin CM, Mendes SDC, Castrillón MR, Steglich R, et al. A case of *Exophiala spinifera* infection in SouthernBrazil: Molecular identification and antifungal susceptibility. Med Mycol Case Rep. 2012;1(1):72–5.
26. Zanini M. Tratamento de cromomicose com criocirurgia e itraconazol sisteḿ ico. Med Cutan Ibero Lat Am. 2012;40(5):168–70.
27. Daboit TC, Stopiglia CDO, Antochevis LC, Heidrich D, Magagnin CM, Vettorato G, et al. Sensibilidade a antifúngicos de isolado clínicode *Fonsecaea pedrosoi* oriundo paciente recidivado após tratamento com itraconazaol. V Simpósio Brasileiro de Microbiologia Aplicada;2011;28-24.
28. De Sousa MDGT, Belda W, Spina R, Lota PR, Valente NS, Brown GD, et al. Topical application of imiquimod as a treatment for chromoblastomycosis. Clin Infect Dis. 2014;58(12):1734–7.
29. Almeida APM, Gomes NMFG, Almeida LM, Almeida JLM. Cromomicose: relato de caso e revisão da literature. Rev Soc Bras Clin Med.2014;12(1):69-1.
30. De Azevedo CMPS, Gomes RR, Vicente VA, Santos DWCL, Marques SG, Do Nascimento MMF, et al. *Fonsecaea pugnacius*, a novel agent of disseminated chromoblastomycosis. J Clin Microbiol. 2015;53(8):2674–85.
31. Azevedo CMPS, Marques SG, Santos DWCL, Silva RR, Silva NF, Santos DA, et al. Squamous cell carcinoma derived from chronic chromoblastomycosis in Brazil. Clin Infect Dis. 2015;60(10):1500–4.
32. Veasey JV, Machado B de AR, Lellis RF, Muramatu LH, Zaitz C. Tumoral chromoblastomycosis: A rare manifestation with typical complementary exams. An Bras Dermatol. 2015;90(6):907–8.

33. Gomes RR, Vicente VA, Azevedo CMPS, Salgado CG, da Silva MB, Queiroz-Telles F, et al. Molecular Epidemiology of Agents of HumanChromoblastomycosis in Brazil with the Description of Two Novel Species. PLoS Negl Trop Dis. 2016;10(11).
34. Ogawa MM, Peternelli MP, Enokihara MMSS, Nishikaku AS, Gonçalves SS, Tomimori J. Spectral Manifestation of Melanized Fungal Infections in Kidney Transplant Recipients: Report of Six Cases. Mycopathologia. 2016;181(5–6):379–85.
35. Cleinman IB, Gonçalves SS, Nucci M, Quintella DC, Halpern M, Akiti T, et al. Respiratory Tract Infection Caused by *Fonsecaea monophora* After Kidney Transplantation. Mycopathologia. 2017;182(11–12):1101–9.
36. Criado PR, Cosenza FD, Junior WB, Ferreira PS. Longitudinal melanonychia due to voriconazole therapy during treatment of chromoblastomycosis. Clin Exp Dermatol. 2018;43(1):75-6.
37. Coelho RA, Brito-Santos F, Figueiredo-Carvalho MHG, Silva JVS, Gutierrez-Galhardo MC, do Valle ACF, et al. Molecular identificationand antifungal susceptibility profiles of clinical strains of *Fonsecaea* spp. isolated from patients with chromoblastomycosis in Rio de Janeiro, Brazil. PLoS Negl Trop Dis. 2018;12(7).
38. Queiróz AJR, Pereira DF, Antônio JR. Chromoblastomycosis: clinical experience and review of literature. Int J Dermatol. 2018;57(11):1351-55.
39. De Andrade TS, De Almeida AMZ, Basano SDA, Takagi EH, Szeszs MW, Melhem MSC, et al. Chromoblastomycosis in the Amazon region, Brazil, caused by *Fonsecaea pedrosoi*, *Fonsecaea nubica*, and *Rhinocladiella similis*: Clinicopathology, susceptibility, and molecular identification. Med Mycol. 2020;58(2):172–80.
40. Diniz YCM, Simões Neto EA, Bomfim MRQ, Conceição PCR, Silva RR, Marques SG, et al. Chromoblastomycosis and Chagas' disease:a case study in the Brazilian Northeast. Braz. J. of Develop. 2019;5(10):21115–30.
41. Belda W, Criado PR, Casteleti P, Passero LFD. Chromoblastomycosis evolving to sarcomatoid squamous cell carcinoma: A case report.Dermatol Reports. 2021;13(2):9009.
42. Melo ED, Morais PM, Fernandes DCL, Rebello PFB. Case for diagnosis. Pruritic erythematosquamous lesion in the auricle. An BrasDermatol. 2020;95(4):521–3.
43. Belda W, Criado PR, Passero LFD. Successful treatment of chromoblastomycosis caused by *Fonsecaea pedrosoi* using imiquimod. JDermatol. 2020;47(4):409–12.
44. Belda W, Casolato ATS, Luppi JB, Passero LFD. Managing chromoblastomycosis with acitretin plus imiquimod: A case report on theimprovement of cutaneous lesions and reduction of the treatment time. J Dermatol. 2021;48(10):1612–5.
45. Carvalho GSM, Calbucci KBCV, Lellis RF, Veasey JV. Presence of hyphae in chromoblastomycosis examinations: an enigma to be solved.An Bras Dermatol. 2021;96(4):490–3.
46. Giraldelli GA, Baka JLCS, Orofino-Costa R, Piñeiro-Maceira J, Barcaui E, Barcaui CB. In vivo reflectance confocal microscopy,dermoscopy, high-frequency ultrasonography, and histopathology features in a case of chromoblastomycosis. 2022;16(3).
